# NK Cells in Mucosal Defense against Infection

**DOI:** 10.1155/2014/413982

**Published:** 2014-08-14

**Authors:** Daria Ivanova, Ryan Krempels, Jennyfer Ryfe, Kaitlyn Weitzman, David Stephenson, Jason P. Gigley

**Affiliations:** Department of Molecular Biology, 6005 Agriculture C Building, University of Wyoming, Laramie, WY 82071, USA

## Abstract

Conventional natural killer cells (NK cells) provide continual surveillance for cancer and rapid responses to infection. They develop in the bone marrow, emerge as either NK precursor cells, immature, or mature cells, and disperse throughout the body. In the periphery NK cells provide critical defense against pathogens and cancer and are noted to develop features of adaptive immune responses. In the tightly regulated and dynamic mucosal tissues, they set up residency via unknown mechanisms and from sources that are yet to be defined. Once resident, they appear to have the ability to functionally mature dependent on the mucosal tissue microenvironment. Mucosal NK cells play a pivotal role in early protection through their cytolytic function and IFN*γ* production against bacteria, fungi, viruses, and parasitic infections. This review presents what is known about NK cell development and phenotypes of mucosal tissue resident conventional NK cells. The question of how they come to reside in their tissues and published data on their function against pathogens during mucosal infection are discussed. Dissecting major questions highlighted in this review will be important to the further understanding of NK cell homing and functional diversity and improve rational design of NK cell based therapies against mucosal infection.

## 1. Introduction

Natural killer cells (NK cells) are a first line of defense against invading pathogens and cancer. Recent studies focused on development and functional diversity of innate immune cells have led to the reclassification of these cell types into a large group known as innate lymphoid cells (ILCs) [1]. This is due to their origin from the common lymphoid progenitor (CLP) but unlike their T cell and B cell counterparts, they do not activate the recombination activation genes (RGA1/2) and do not undergo antigen receptor rearrangement. There are three main groups, Group 1, of which conventional NK cells are members, Group 2, and Group 3. Each grouping is based on the functionality and transcriptional regulation of cell type development. NK cells are members of group 1 ILCs due to their ability to produce IFN*γ* and be cytolytic. Their activation and function rely on recognition of pathogen-infected cells through activating receptors (KIRs in humans and Ly49 in mice) and proinflammatory cytokines. NK cells can also regulate immunity. During systemic infections they produce IL-10 and with high viremia can target DCs and T cells, thus modifying immunological memory [2–5]. As such, NK cells have many roles, in protection, in helping to maintain immune homeostasis, and in long term immunity.

NK cells are found in many tissues. This includes bone marrow (BM), blood, liver, thymus, and spleen. Mucosal sites that harbor NK cells include the lung, the small and large intestine and colon of the gastrointestinal tract (GI), and the uterus, cervix, ectocervix, and vagina of the female reproductive tract (FRT). Much of how they gain access to these sites and provide function (protection, immunoregulation) is just beginning to be understood. The review focuses on recent work and the current understanding of the regulation of mucosal tissue residency of NK cells and NK cell functional importance at mucosal sites relevant to both mouse and human systems. We will not address ILC2 and ILC3 populations as those have been reviewed elsewhere [6, 7].

## 2. NK Cell Development

In humans and mice, NK cells develop from the common lymphoid progenitor (CLP) in the bone marrow [8]. CLPs in the mouse BM differentiate into a pre-NK precursor (pre-NKP) with a phenotype of Lin^−^ CD117^−^CD127^+^ and express some NK cell specific receptors including NKG2D and 2B4 (CD244) and negative for classical NK cell markers NK1.1 and CD49b. Pre-NKP then express the *β*-chain receptor for IL-15 (CD122) and CD11b and are now defined to be NK precursors (NKP). IL-15 is a cytokine required for their development as shown by IL-15 KO mice, which predominantly lack NK cells [9]. Interestingly, infection of IL-15 KO mice with the protozoan parasite* Toxoplasma gondii* or IL-15 KO, IL-15R*α* KO, and RAG2/IL-2R*γ* KO mice with MCMV infection results in rapid expansion of NK cells [10, 11]. These studies support IL-15 as an important cytokine for promoting NK cell development in the absence of infection. However, they demonstrate that other non-*γ*-chain cytokines such as IL-12 during infection can stimulate NK expansion and activity independent of IL-15. Once CD122 is expressed, NKPs now become responsive to IL-15 and develop further into immature NK cells (iNK) marked by CD27^lo^  CD11b^lo^ and the progressive expression of activating and inhibitory receptors. Murine NK cells have been further defined based on the expression of CD27 and CD11b into 4 stages of maturation that correspond with their production of inflammatory cytokines and cytolytic activity [12, 13]. After the iNK stage they progress in phenotype from the CD27^lo^  CD11b^lo^ to CD27^hi^  CD11b^lo^ then from CD27^hi^  CD11b^hi^ to CD27^lo^  CD11b^hi^.

Human NK cells develop from hematopoietic stem cells (HSCs) through a CLP as in mice with differentiation points designated as development stages (stages 1–5) [8]. Stages 2 and 3 cells are CD56^−^ and CD94^−^ and become responsive to IL-15. Stages 4 and 5 cells acquire the expression of the human classical NK cell marker CD56 and are now considered to have differentiated into the NK cell phenotype. Stage 4 cells are CD56^bright^ Fc*γ*RIII^−^ (CD16^−^), produce high levels of cytokines, have low cytolytic capabilities, and are considered immature. Stage 5 cells are CD56^dim⁡^ CD16^+^, are both cytolytic and capable of producing high amounts of IFN*γ*, and are considered mature.

## 3. NK Cell Phenotypes and Migration to Mucosal Sites

NK cells are present systemically in bone marrow, secondary lymphoid structures, peripheral nonlymphoid organs, and blood. In lymph nodes, the majority of NK cells are considered immature and are CD56^bright^CD16^−^ in humans and CD27^hi^CD11b^lo-med^ are in mice [8, 14]. In nonlymphoid organs, such as gut and lung, resident conventional NK cells have varying phenotypes based on maturation and function. The factors governing the phenotype and function of resident NK cells at these sites are unknown. However, NK cell diversity at these different sites is likely dependent upon the integration of environmental signals to promote their needed activity. Additionally, the ultimate source (blood, BM, or lymph node (LN)) of resident NK cells in these organs is not well described. While human and murine NK cells undergo the above-mentioned developmental stages, once they exit the BM could migrate to either the blood stream or into secondary lymphoid structures. Whether immature NK cells from LN or more mature cells from the blood seed peripheral tissues during the steady state is unclear. Recently, liver NK cells were demonstrated to be distinct in development from thymic and splenic NK cells [15]. Therefore mucosal tissue specific NK cells could also differentiate* in situ* rather than be seeded by LN or peripheral blood precursors. Regardless, there are several necessary steps for this post-bone-marrow phase of NK cell development and function at mucosal sites. These steps include migration, changes in phenotype, education, and maturation. In addition to what controls homing of NK cells to mucosal tissues, the mechanisms behind how mucosal NK cells adjust to their resident environments are unclear and will be important to dissect.

The current model of NK cell development and migration suggests that NK cells likely emerge from BM as a mix of mature and immature cells. Immature cells mature and acquire organ specific phenotypes in the extramedullary tissues including secondary lymphoid tissues and liver [14–18]. Mature NK cells circulate to different tissues and are then modified by tissue microenvironments via cytokine milieu, growth factors, or chronic inflammation [7, 19]. Migration from BM to a specific tissue is a complex and critical first step to establishing residency. This process is likely different for each nonlymphoid tissue and has not been well described. A good example of the complexity of this process is how specific CD8 T cell populations are recruited to the gut to become intraepithelial lymphocytes (IELs). Intestinal mucosa homing of IELS requires a priming event near the tissue itself or in the mesenteric lymph node (MLN). Priming is dependent upon an interaction with mucosal CCR7^+^ CD103^+^ DCs and acquisition of *α*4*β*7 integrin and CCR9 expression [20–23]. Given the intense research in this area, the gut IEL model for tissue homing may be a useful starting point to base mechanistic studies on NK cell recruitment to mucosal nonlymphoid tissues.

### 3.1. Resident NK Cells of the Lung Mucosa

**Figure 1:**
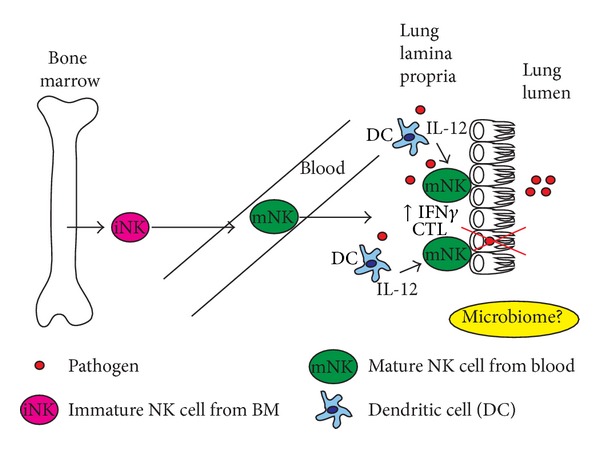
Lung mucosa NK cells. NK cells found in the lung during the steady state exhibit a mature phenotype being CD27^lo^CD11b^hi^ in mice and CD56^dim⁡^ CD16^+^ in humans suggesting that after development in the bone marrow immature (iNK, pink) and or mature (mNK, green) NK cells found in the blood home to the lung and may not require a specific signal or cell-cell interaction in a secondary lymphoid organ to migrate. Lung microbiome could impact NK cell development and/or function. During infection by a pathogen (red circle), dendritic cells in the lamina propria of the lung are triggered via pattern recognition receptors (PRRs) to produce inflammatory cytokines including IL-12 and IL-23. This in combination with recognition of infected cells by activating receptors (Ly49 mouse, KIR human, NKp both) on NK cells results in activation of resident NK cell populations to produce IFN*γ*, IL-17, and be cytolytic (CTL).

10% of total lung resident lymphocytes are NK cells. This is a higher NK cell number than other nonlymphoid organs highlighting their importance in this tissue [24]. They are primarily found along with other lymphocytes in the lung epithelium and vascular tissues [24, 25]. In humans, lung resident NK cells are CD56^dim⁡^ CD16^+^ and mostly NKp46+ similar to NK cells in the blood suggesting a mature phenotype capable of being cytotoxic and producing cytokines [26]. In mice the more mature NK cell phenotype is found in the lung epithelium, which is CD27^lo^  CD11b^hi^, very similar in function to the human subset. In the naïve state, these two phenotypes of NK cells in both human and mouse compose 80–90% of all NK cells present in the lung tissue [13, 25]. The maturation status of most lung NK cells resembles those from blood. However a recent study identified a population of NK cells in the lung capable of being further differentiated [27]. This study demonstrated that, unlike bone marrow precursors, the lung precursor cells when cultured* in vitro* expressed more Ly49 receptors. These results suggest that both mature and immature NK cells are present in the lung and that the murine lung microenvironment could condition NK cells separately from the bone marrow. In humans, other than classical NK cell marker phenotype (CD56 and CD16) approximately 30% of lung resident NK cells express KIRs including KIR2DL2, KIR2DL3, KIR3DL1 and KIR2DS2. Nearly 80% of the CD56^bright^ population expressed CD94 similar to the phenotypes found in peripheral blood [26, 28]. Murine and human lung microenvironments may differ in their ability to modify resident NK cells. In humans support for tissue specific microenvironment conditioning of NK cells comes from studies of pulmonary sarcoidosis and non-small-cell lung cancer [26, 28]. NK cells express less KIR in bronchiolar lavage fluid (BALF) in these situations than in the naïve state. Interestingly, peripheral blood NK cells also had lower KIR expression. Whether or not the change observed in BALF was from migrating cells is not known. The observations in BALF support a role for tissue specific microenvironment conditioning of NK cells. However, the more mature phenotype of resident NK cells in the lung is suggestive of a blood origin (Figure 1). Currently, much is still not known about the mechanisms behind these changes and whether in the healthy state tissue resident human NK cell phenotypes can be modified by the lung.

### 3.2. Resident NK Cells of the Gut Mucosa

**Figure 2:**
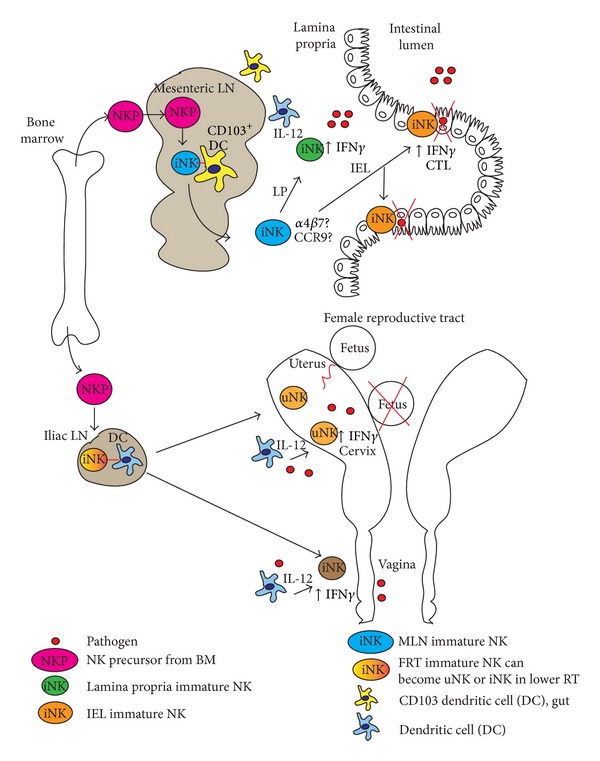
Gut mucosal and female reproductive tract NK cells. NK cells found in the gut mucosa and FRT during the naive state exhibit an immature phenotype being CD27^med-hi^CD11b^hi^ in mice and CD56^bright^CD16^−^ in humans and resemble those NK cells found as residents in secondary lymphoid organs such as mesenteric lymph node (MLN) for the gut (top) and Iliac lymph node (ILN) for the FRT (bottom). This may suggest that gut and FRT resident NK cells are derived from NK precursors (NKP, pink), which migrate from bone marrow to lymph nodes (LN), where they differentiate into immature NK cells (iNK, blue cell in MLN, orange-red transition cell ILN). Dendritic cells (DC) draining from the lamina propria of the gut (CD103^+^) or DC from the FRT to the LN interact with immature NK cells and imprint the tissue address (*α*4*β*7/CCR9 for gut mucosa) that permits iNK cell homing to the correct tissues. In gut, NK cells can be IELs (orange cells) or in the LP (green cell) and respond to infectious pathogen (red circles) activated DCs and cytokine milieu in the tissue microenvironment becomes activated to produce IFN*γ*, IL-17, and/or CTL activity. In FRT, uterine NK cells (uNK, orange cell) have to balance help with fetal implantation via trophoblast recruitment, vascularization, and tolerance with ability to respond to pathogens. If stimulation of NK cells in response to a pathogen is high enough, IFN*γ* is expressed and may result in loss of pregnancy. Vaginal NK cells (iNK, brown) play an important role in containing invading pathogens via IFN*γ* production.

Of the three innate lymphoid cell populations found in the gut, conventional NK cells of the gut are classified to belong to the ILC1 subset [1]. NK cells are present in all gut tissues (small intestine, large intestine, and colon) as cells of the IEL and LP compartments. They can also be found in smaller numbers in Peyer's patches (PP) and MLNs. Unlike the lung, gut mucosal NK cells in humans are predominantly CD56^bright^ with few that express CD16 indicating that they may be similar to immature NK cells found in secondary lymphoid structures [29–31]. NK cells in the murine intestinal mucosa also appear immature. IEL and LP NK cells of naïve mice are CD27^hi^CD11b^med^ and CD27^lo^CD11b^lo^, respectively [32–34]. Further support for an immature phenotype for both murine and human gut NK cells is evident from functionality. Resident human CD56^bright^ and mouse CD27^hi^ NK cells produce large amounts of the proinflammatory cytokine IFN*γ* and exhibit low cytolytic activity when tested* in vitro* [19, 31]. After infection, significant changes occur in NK cell frequencies in the small intestine and lamina propria. Whether peripheral NK cell infiltration occurs or whether there are phenotypic changes in resident NK cell populations has not been investigated. Infiltration of peripheral blood NK cells probably occurs albeit to a lesser extent and the NK cell dependent response may rely more on an NK cell interaction with activated gut mucosal DCs in the MLN and/or expansion of activated LP NK cells at the site of infection (Figure 2). However, given the diversity of gut mucosal ILC populations with wide ranging function, determining the level of infiltration of new cells may be very difficult.

### 3.3. Resident NK Cells of the Female Reproductive Tract

The FRT is populated with NK cells from the uterus to the vagina. NK cells play an important role in not only host defense against pathogens, but also successful vascular remodeling of the uterus during pregnancy and fetal implantation [35, 36]. In humans, FRT NK cells resemble an immature phenotype of NK cells similar to that found in the digestive tract and are CD56^bright^CD16^−^ [37]. NK cells found in the uterus are phenotypically distinct from others in the FRT being CD9^+^ and chemokine receptor like −1^+^ (CMKLR1). CD9 is a tetraspanin family member important for cell adhesion and migration and CMKLR1 is a receptor for chemerin, which promotes migration to the decidua and vascular remodeling during pregnancy [38, 39]. They are also positive for KIR molecules (KIR2DL4) and CD94/NKG2A. Uterine NK cells exhibit functional characteristics of immature NK cells having high cytokine production and low cytotoxic potential. Murine uterine NK cells resemble their human counterparts as having a more immature phenotype being CD27^Med^  CD11b^Hi^ [36]. They also express Ly49 receptors including Ly49D, H, C, I, G, and A. They are different from human uterine NK cells in that they exhibit lower cytolytic activity and are more important in contributing to vascular remodeling for proper fetal implantation [40].

Vaginal resident NK cells in humans are different from their uterine counterparts in that they are CD16^+^ and CD94^−^ [39]. They also had a high potential of producing inflammatory cytokines including IFN*γ* and lower cytotoxic potential. They express CD56 to a similar level as immature NK cells and are important in early responses to infection. In mice, vaginal NK cell maturation phenotype based on CD27 and CD11b is unclear. Yet they make up approximately 20% of total leukocytes present and are capable of high level IFN*γ* production when stimulated* ex vivo* [41]. Given their importance, a more thorough analysis of their phenotype would be interesting and informative to pursue.

### 3.4. NK Cell Homing to Mucosal Sites

The mechanisms behind establishment of resident NK cells at mucosal sites are still not well described. Chemokines most likely play a very important role in this process and mucosal associated NK cells express a vast array of chemokine receptors including, CXCR1, CCR2, CCR7, CCR5, and CX3CR1 [42]. Additional factors are important in homing of NK cells to these sites including chemerin for female reproductive tract resident cells and sphingosine-1 phosphate family member S1P5 [43]. Given the diversity of expression of these receptors on NK cells, each tissue could regulate which cells migrate to which organ.

An intriguing model behind how NK cells set up residency in different tissues has been recently proposed [8, 43, 44]. This model suggests that, unlike secondary lymphoid tissues and blood, NK precursor cells migrate from the BM into the blood then migrate further to different sites in the body including mucosal tissues. Once they have migrated, organ specific environments influence their development. Likely contributors include estrus cycling hormones, inflammatory milieu such as IL-15 by somatic cells, TGF*β*, IL-10, and the resident microbiomes. Factors from these sources could induce NK precursor differentiation to attain different levels of maturation and education. New data on tissue specific difference in NK cell differentiation and studies investigating whether NK cells can be differentiated from tissue specific precursors may support the hypothesis that NK precursor cells seed peripheral organs and that they differentiate independent of the bone marrow [27, 45]. This would allow these tissue resident NK cells to be “educated” to have very specific functions important at sites to which they migrate [8].

An alternative hypothesis is possible. In certain mucosal tissues, especially those with established microbiomes, such as gut, FRT, and recently described in the lung [46], NK cells may require a priming event from draining APC populations. These APCs program LN NK cells in mucosal associated lymphoid tissue (MALT) to migrate to a specific site (Figure 2). MALT includes lymph nodes such as the mesenteric lymph node (MLN). This hypothesis is supported by the conserved phenotypic characteristics of gut and female reproductive tract NK cells. They more closely resemble immature cells by being CD56^med-bright^ CD16^+^ and CD27^+^ CD11b^+^ with higher cytokine production in humans and mice, respectively. As mentioned above, lung NK cells resemble a more mature phenotype and could come directly from the peripheral blood (Figure 1). Despite the lung NK cell phenotype, the presence and the effect of the lung microbiome on the development of asthma and chronic inflammation suggest that NK cells could also be modified* in situ*. Regardless, support for the MALT APC priming hypothesis could be taken from the process of T cell (IEL) recruitment into the intestinal epithelium. Interactions between gut mucosal T cells and CD103^+^ DCs via CRTAM1 in both the steady state and during infection are required for recruitment [47]. IELs during pathogenic situations require IFN*γ* producing mucosal DCs to be primed to home to the gut [20]. Interestingly, a recent report demonstrates that small intestine IELs emerge from the thymus as recent thymic emigrants which express differing levels of the gut homing integrin *α*4*β*7 [22]. *α*4*β*7 is required for IEL trafficking to the gut [21]. Since NK precursors and other ILC populations in secondary lymphoid tissues express varying levels of this integrin, it may be possible that an NK-DC interaction is a requirement for immature NK cells to be signaled to home to mucosal sites. Altogether, this is a possible mechanism important for homing that would be interesting to explore.

## 4. Infection and Mucosal NK Cells

During mucosal infections of humans and mice, NK cells are recruited to sites of infection and play an important role in immune defense [6, 48]. Soon after infection and ensuing inflammation (type I IFN and IL-12), resident NK cells respond, produce IFN*γ* and TNF*α*, and become cytotoxic [49]. Additional NK cells are recruited from the periphery within a few days of these events and contribute to these responses. As mentioned before, although the cytokine IL-15 is required for development and the expansion of developing NK cells, other cytokines and signals can cause NK cells to expand and respond to infection [11]. Investigation into how NK cells in IL-15 KO or IL-15R*α* mice increase in number with MCMV infection demonstrated that IL-12 promotes their expansion and activation. Additionally, stimulation of NK cells through the activating receptor Ly49H via m157 of MCMV also contributed to this response. Therefore the cytokine milieu present in the different mucosal tissues in addition to activating signals stimulated that by diverse pathogens help NK cells respond to infection. Peripheral NK cell migration occurs in all mucosal tissues during bacterial, fungal, viral, and parasitic infection [6, 37, 43, 44]. Recruitment to inflamed tissues is largely dependent upon chemokines and the ligation of their cognate receptors on NK cells. Once activated conventional NK cells at these sites are vital for initial containment of pathogens and preventing their systemic spread.

### 4.1. Role of NK Cells in Pulmonary Infections

The lung is a site of entry for viral, bacterial, and fungal infection. Of all nonlymphoid tissues, the lung contains the largest number of conventional NK cells. Humans and mice with genetic deficiencies resulting in loss of NK cells are more susceptible to pulmonary infections [50–52]. Many viruses including influenza, coronavirus (severe acute respiratory syndrome (SARS), middle eastern respiratory syndrome (MERS)), herpes, and respiratory syncytial virus (RSV) infect via the airways. NK cells were demonstrated early to respond to influenza infection in the lungs of humans through their production of IFN*γ* [53]. Cytolytic activity of these cells is also important for control of influenza infection including pandemic H1N1 influenza [54]. NK cell CTL activity in response to influenza is mediated through antibodies and ADCC or via the recognition of viral haemagglutinin by the natural cytotoxicity receptors NKp46 [55]. In mice infected with influenza A (PR8), infiltrating NK cells are CD27^low^CD11b^hi^ and appear to be of a mature phenotype [56]. In line with this, they also have greater cytolytic activity than IFN*γ* production suggesting that they could be NK cells migrating in from the blood or replicating* in situ* [57]. In addition to IL-12, IL-15 has been shown to play a role in NK cell responses in the lung [52, 58, 59]. IL-15 in complex with IL-15R*α* can help in recruitment and activation of NK cells during rhinovirus infection and IL-15 when blocked with an antibody appears to prevent NK cell recruitment into the BAL during influenza infection in mice [58, 59]. NK cell protective function in the lung against extracellular* Staphylococcus aureus* also appears to require IL-15 [52]. IL-15 likely contributes to the expansion and synergizes with IL-12 and IFN*α* for proper activation of NK cells in the lung. Overall, it appears that, in the mouse model, NK cells are required for survival against influenza infection [60].

NK cells functioning to control influenza can also cause severe pathology in the lungs [56]. In mice given a high dose of influenza, depletion of NK cells resulted in better outcome against infection. A reason behind these differences could be attributed to the proinflammatory cytokines produced during initial viral encounter that help NK cells respond. IL-12 and type I IFNs stimulate NK cells to produce IFN*γ* [61]. However, high production of these cytokines in response to pulmonary infection can lead to overproduction of IFN*γ* resulting in tissue destruction.

NK cells play a role in early immunity and control of lung fungal and bacterial infections. NK cells help in early control of* Cryptococcus neoformans* and aspergillosis through their cytolytic activity and IFN*γ* production [62–65]. By producing IFN*γ* these cells play a critical role in control of several bacterial infections including* Staphylococcus aureus*,* Klebsiella pneumonia,* and* Legionella* infections [52]. In the case of* Klebsiella*, NK cell production of IL-22 may also be important in establishing immunity. In response to* Mycobacterium tuberculosis* (Mtb) infection, IFN*α* *β* and IFN*γ* are critical for control of the bacterium, the former via restriction of macrophage infiltration into the lung and the latter through inhibition of microbial growth [66]. As with many infections, NK cells respond to both type I IFN and IL-12 during Mtb infection resulting in their production of IFN*γ* and are critical for early survival in the mouse model as demonstrated in RAG2−/− common-*γ*-chain−/− and IL-12p40−/− mice [67]. However, depletion of NK cells has shown that, like some fungal models, NK cells can contribute to early control of Mtb but are not required for long term survival against infection [68].

Overall, NK cells are very important for early control of pathogens in the lung. The majority of this protective response is governed by the production of IFN*γ* by these cells and can be regulated via IL-12 and IFN*α*/*β* provided by myeloid populations. Activation is also aided by the integration of activation signals through activating receptors on the cells. NK cell can also be pathogenic in the lung during infection from overproduction of IFN*γ*. Questions still remaining that would be interesting to address are how to boost NK cell responses to this infection without causing overt tissue pathology and also what is the advantage of having NK cells present in the lung to promote this early control. Are NK cells more important for immune stimulation or regulation or do they have bifunctional roles as a plastic cell able to respond to the environment? Further investigation of the biology of these cells in the lung is needed to answer these questions.

### 4.2. Role of NK Cells in Gastrointestinal Tract Infections

As mentioned above, ILC populations in the GI tract are very diverse and have many roles from innate immune control of pathogens to regulation of autoimmunity [7, 8, 44]. IFN*γ* producing conventional NK cells helps to control many infectious pathogens in the gut. Although NK cells appear to be dispensable for* Listeria monocytogenes *infection in mice [69], in a rat model of infection, they were essential in they were essential for early control of the bacterium [70]. Investigation of* Salmonella *infection in mice demonstrated that IL-15 and NK cells were required [71]. IL-15 KO mice had greater bacterial burdens than WT animals. Depletion of NK cells also resulted in the great colonization of the murine gut with* Salmonella*. Despite previous studies where IL-15 KO mice are still able to mount a robust NK cell response during viral and parasitic infection due to IL-12 production this did not appear to occur during bacterial infection. This difference may highlight a difference in gut NK cells or variation in gut NK cell responses to different pathogens [10, 11]. Recently, LP NKp46+ NK1.1+ ILCs were shown to contribute to gut pathology and ileitis during* Toxoplasma gondii* infection [72]. Their function relied on IL-15 and resulted in CCL3 dependent recruitment of inflammatory monocytes into the lamina propria. Gut NK cells therefore may have several functions, not only in controlling infections directly, but also in the coordination and regulation of immune responses in the intestinal mucosa.

NK cell IFN*γ* is also important in control of gut* Citrobacter rodentium* and* Yersinia enterolytica* infections [73–75]. IFN*β* production from macrophages was critical for this response. Interestingly, TRIF signaling downstream of TLR4 was required for this macrophage induced NK cell activity. NK cells in the GI tract are involved in control of parasitic infection. NK cells production of IL-17 in response to* Toxoplasma gondii* infection in the gut was dependent upon IL-6 [76]. In another model, NK cell dependent and independent IFN*γ* was required to control* Cryptosporidium parvum *infection in mice [77, 78]. NK cells in humans are also important for innate control of gut mucosal infections. In HIV patients, individuals who are known as spontaneous controllers have greater numbers of IEL associated NK cells than those who are classified as nonresponders [79]. Control of HIV in humans and also SIV in macaques could be due to NK cell derived IL-17 and IL-22 production in IELs and dependent upon an interaction with gut mucosal CD103+ dendritic cells [80]. NK cells may contribute to HIV pathogenesis early in infection as tested with an SIV model. This was shown when *α*4*β*7 was blocked by antibody treatment in macaques [81]. Trafficking of NK cells blocked with this treatment reduced SIV dissemination and viral burdens. NK cell activation itself may also contribute through recruitment of CD4 T cells providing the virus with more replicative niche to survive. However, later NK cells could play a substitutive role in protection. Since *α*4*β*7 was required for NK cell recruitment in this model, these results give support to the hypothesis that, in the gut, NK cell homing depends upon an interaction with mucosal dendritic cells (Figure 2, top). Overall NK cells in gut mucosa play an important role in protection against orally acquired infections and further dissection is needed to understand how to take advantage of this cell type to optimize protection.

### 4.3. Role of NK Cells in Female Reproductive Tract Infections

There is a wide distribution of NK cells in mice and humans throughout the female reproductive tract, from the uterus, endometrium, and cervix to the ectocervix and vagina [39, 82]. The resident cells in these tissues are all capable of producing IFN*γ* and cytolytic activity through ADCC. The importance of IFN*γ* was noted early in response to vaginal infection of mice with HSV-2 where it was shown to help in resolving this infection in mice [83]. Subsequent studies in mice revealed that a major source in the vaginal mucosa for this cytokine was NK cells [84]. This was demonstrated using RAG2−/− common–chain−/− mice, which lack T, B and NK cells and RAG2−/− mice, which lack only T and B cells. RAG2−/− common–chain−/− mice were 100 fold more susceptible to infection than RAG2−/− mice. Although it was shown later on that IL-15 could stimulate an anti-HSV-2 response independent of NK cells [85], as in gut mucosa infections, IL-15 was important in stimulating NK cell protective responses. Recruitment of NK cells was also important for the ability of NK cells to protect against vaginal HSV-2 infection. CCR5−/− mice were more susceptible and had higher viral loads in the CNS after vaginal infection most likely due to impaired NK cell trafficking to the site of infection [86]. Using a RAG2−/− common-*γ*-chain−/− humanized mouse model, human NK cells were observed to be recruited into the genital tract of female mice infected with HSV-2 suggesting that NK cell innate protection could be important in humans as well [87]. Further evidence of a potential role for NK cells in human reproductive tract comes from SIV-macaque models where treatment of SIV infected animals with IFN*α* and IL-12 resulted in enhanced activation and ADCC function of CD56^+^CD16^+^ cells in the vaginal mucosa [88]. Much investigation is still needed to understand NK cell responses in the vaginal mucosa as they do appear to be important for protection, but they can also contribute to disease pathology as seen in early HIV infection.

Uterine NK cells play two important roles in that they are required for tissue reorganization, vascularization, implantation of the fetus, and tolerance. At the same time they play a role in providing fetal protection during infection (Figure 2, bottom) [89]. What regulates the balance between tolerance and protection is not clear. Infection with* Listeria monocytogenes* results in placental colonization [90]. NK cells tested for their ability to prevent this colonization in mice were shown to not have an impact. Although IL-15 was required for uterine NK cell development and their recruitment to this site in the naïve state, a lack of IL-15 did not prevent the production of IFN*γ* early during* Listeria *infection. Therefore, IL-15 in the uterus appears to play a more important role in NK cell recruitment but less of a role in early NK cell control of infection [91]. Other factors may be required to regulate their responsiveness to infection in this tissue. Human uterine NK cells did not respond to direct TLR triggering but required an interaction with TLR stimulated accessory cells from the uterus to produce IFN*γ* [92]. Therefore, pathogen stimulation could cause an imbalance towards inflammation rather than tolerance at the maternal-fetal interface. This is supported by a study investigating* Toxoplasma gondii* infection induced abortion in mice [93].* T. gondii* induces a potent Th1 response including NK cell production of IFN*γ*. Parasite infection is also known to cause spontaneous abortion in the first trimester of pregnancy. Infection of pregnant wild type mice in this study resulted in fetal resorption while infection of pregnant IFN*γ*R−/− mice had 50% reduction in effect. Surprisingly, this was shown to be independent of NK cells. However, recent studies using decidual NK and trophoblast cells from humans have demonstrated that activity of these NK cells is enhanced upon* T. gondii* infection [94]. This increase in activity was due to the increased expression of NKG2D by decidual NK cells after* in vitro* stimulation with* Toxoplasma *[95]. NK cells may sense their environment and be tuned commensurate with what functions are needed. This possibility is supported by the theory of NK cell reeducation as what occurs when NK cells develop a memory phenotype [96]. Since uterine NK cells recruited in the steady state are less mature, as noted by their surface marker phenotype, the cytokine/hormone milieu changes the functional phenotype needed for successful pregnancy or immune protection develops* in situ*. Another possibility is that new NK cells from the periphery could be recruited and contribute to pathology. Pathogen stimulated that APCs migrating to the draining lymph node activate newly recruited NK cells that are then driven to protect. Much needs to be addressed in regard to the mechanisms behind FRT NK cell function as there is relatively little known.

## 5. Conclusions

Conventional NK cells are found in all mucosal tissues and play an important role in first line of defense against bacterial, fungal, viral, and parasitic infections. Significant questions still remain in regard to (1) how they gain entry into each of the specific tissues, (2) what sources of NK cell precursors provide resident NK cells in different tissues, and (3) how their diverse functional responses are regulated in different mucosal tissues. For mucosal tissue resident NK cells in the naïve state, lung NK cells may represent a more mature phenotype and could come from the blood stream. Gut mucosal and FRT NK cells may be derived from NK precursor or immature NK cells after receiving initial address directions and programming from APCs in secondary lymphoid structures. During infection, resident mucosal tissue NK cells respond primarily through IFN*γ* production, which contributes directly to early control of pathogens. Inflammation (IL-12, IFN*α*/*β*, IFN*γ*, and chemokines) at the site of infection then results in expansion of resident cells and recruitment of peripheral NK cells which provide additional protection. Resident NK cells can change their functional phenotype based on the intensity of the inflammatory signals resulting in pathology in different tissues. Further studies will be important to elucidate mechanisms behind all of these processes and lead to the development of NK cell dependent therapies as well as vaccine approaches that could be useful in not only infectious disease, but also autoimmunity and cancer.
